# Adapting Minds: Exploring Cognition to Threatened Stimuli in the Post-COVID-19 Landscape Comparing Old and New Concerns about Pandemic

**DOI:** 10.3390/brainsci14070711

**Published:** 2024-07-15

**Authors:** Giuseppe Forte, Francesca Favieri, Ilaria Corbo, Giovanna Troisi, Giulia Marselli, Barbara Blasutto, Renato Ponce, Enrico Di Pace, Viviana Langher, Renata Tambelli, Maria Casagrande

**Affiliations:** 1 Department of Dynamic, Clinical Psychology and Health Studies, “Sapienza” University of Rome, 00185 Rome, Italy; francesca.favieri@uniroma1.it (F.F.); ilaria.corbo@uniroma1.it (I.C.); viviana.langher@uniroma1.it (V.L.); renata.tambelli@uniroma1.it (R.T.); maria.casagrande@uniroma1.it (M.C.); 2 Department of Psychology, “Sapienza” University of Rome, 00185 Rome, Italy; giovanna.troisi@uniroma1.it (G.T.); giulia.marselli@uniroma1.it (G.M.); barbara.blasutto@uniroma1.it (B.B.); renatojavier.ponceguerrero@uniroma1.it (R.P.); enrico.dipace@uniroma1.it (E.D.P.); 3 Department of Experimental Psychology & Mind, Brain and Behaviour Research Centre (CIMCYC), University of Granada, 18011 Granada, Spain

**Keywords:** COVID-19 pandemic, COVID-19-related stimuli, emotional Stroop task, attentional bias, PTSD COVID-19 related

## Abstract

The global population has been significantly affected by the pandemic in terms of physical and mental health. According to transactional theory, individuals have undergone an adaptation process influenced by cognitive control abilities. Emotional responses to COVID-19-related stimuli may interfere with top-down attentional processes, thereby hindering adaptation. This study aimed to investigate the impact of COVID-19-related stimuli on attentional processing and to determine whether psychological factors could modulate these effects. A sample of 96 healthy undergraduate students participated in an emotional Stroop task in which they were presented with a series of stimuli, including both neutral and negative COVID-19-related as well as non-COVID-19 stimuli. COVID-19-related PTSD, as an index of distress (PTSS), and trait anxiety were evaluated. Results showed that participants were more accurate in identifying COVID-19-related stimuli compared to non-COVID-19 stimuli. Being female and having higher retrospective PTSS scores related to COVID-19 were predictive of faster reaction times for both neutral and negative COVID-19-related stimuli. This heightened attentional bias toward COVID-19-related stimuli suggests that individuals may be more sensitive to stimuli associated with the pandemic. The results suggest that the association between COVID-19 stimuli and attentional biases extends beyond emotional valence, being retrospectively influenced by mental health, suggesting potential pathways to future mental health challenges.

## 1. Introduction

Despite the passage of time since the 2020 outbreak of COVID-19, it is clear that the consequent pandemic has had a profound impact on the global population. A lasting effect on both physical and mental health is evident in many individuals worldwide [1,2]. A substantial body of the literature has documented a dramatic increase and exacerbation of depression, sleep disturbances, and post-traumatic stress symptoms compared to the pre-COVID-19 era across various populations [3,4]. These conditions were observed not only in patients who contracted the illness but also in the general population. These effects have been attributed to exposure to COVID-19-related stressors, which include social isolation, changes in interpersonal relationships, and health-related concerns such as uncertainty about the pandemic’s duration and fear of infection and its consequences [5,6,7,8]. In this regard, prolonged exposure to COVID-19-related content has also been shown to impact cognitive functioning, particularly concerning the monitoring and control of behaviors [9]. This is supported by evidence highlighting the detrimental effects of persistent worry, anxiety, and distress over an extended period [10,11,12].

In line with these topics, researchers continue to investigate the ongoing role of the COVID-19 pandemic on well-being, given the emergence of new concerns and global crises since the pandemic. The objective is to understand how this period continues to impact our mental health, cognition, and behaviors [12,13].

According to transactional theory [14,15], the process of individual adaptation to significant sources of stress involves appraisals of different degrees of cognitive control. While responses to trauma-related stressors vary widely among individuals [16], it is plausible that such exposure may not only shape our immediate adaptive responses but also influence long-term reactions to stimuli perceived as threatening through brain and cognitive plasticity. This perspective is particularly relevant within the context of the pandemic, given the direct and indirect consequences of the spread of the virus (i.e., social lockdown, social distancing, isolation, changes in daily habits [17,18,19]), which have been confirmed as distressors and causes of post-traumatic symptoms (PTSSs) [20,21,22]. Consequently, emotional responses to pandemic-related stimuli, influenced by both environmental contexts (explicit factors) and individual backgrounds (implicit factors), may interfere with top-down cognitive processes, such as attentional control. This influence could potentially continue to resonate, affecting our lives and well-being to varying degrees over time [23]. Accordingly, examining the processing of pandemic-related stimuli is crucial for understanding the ongoing impact of the pandemic experience. In this context, the concept of attentional bias becomes valuable. The term “attentional bias” refers to the tendency to focus more on negative information, indicating a heightened allocation of attention towards negative stimuli compared to neutral stimuli [24,25,26]. Recent studies have investigated the impact of exposure to negative information about the novel coronavirus (COVID-19) on attentional bias as a result of exposure to negative information about COVID-19 (i.e., news or rumors), which in turn affects levels of anxiety and health outcomes [27,28]. These findings highlight the importance of focusing on these aspects. A study by Cannito and colleagues [29] reported an attentional bias toward virus-related stimuli associated with increased health anxiety during the initial phases of the emergency. Similarly, a recent study by Rubin and Evans [30] found that individuals experiencing grief due to the COVID-19 pandemic showed dysregulation of affective attentional processes, influencing the processing of COVID-19-related stimuli [30]. Building upon these premises and aiming to investigate the potential long-term effects of the traumatic experience of the pandemic, this study explored the impact of stimuli related to the pandemic on attentional control.

Based on previous studies on attentional bias towards threatening stimuli [28,29,31] and considering the highly negative impact of the pandemic experience, this study hypothesized that COVID-19-related stimuli affect attentional processing, given that they are emotionally salient and have a negative valence. Moreover, it was expected that psychological dimensions that have been demonstrated to modulate emotional attentional processing (e.g., anxiety [25]) would play a role in influencing the processing of these stimuli.

## 2. Materials and Methods

### 2.1. Participants

The participants were recruited via the dissemination of banners advertising this research through the main social media of the “Sapienza” University of Rome. The G-Power analysis (es = 0.20, alpha = 0.05, beta = 0.80; tested for planned analysis) indicated an adequate sample size of at least 75 respondents. Accordingly, we planned to recruit 100 participants to eventually include a suitable number of participants reporting all the data, reducing the risk of missing value. Of the recruited volunteers, 96 respondents (women: 58.3% of the sample; age range: 19–35; mean age: 24.1; SD = 3.02) met the inclusion criteria of this study (i.e., no diagnosis of COVID-19, no psychopathological or medical conditions, no current medications) and participated in this study. The main characteristics of the sample are shown in Table 1.

### 2.2. Measures

The demographic and COVID-19-related information were collected through two sections of a survey. The first section gathered demographic details such as gender (male or female), age, education, and medical and psychopathological history in order to assess the individual’s eligibility to participate in this study. The second section focused on the assessment via self-questionnaires of the variables of distress and anxiety.

#### 2.2.1. Post-Traumatic Stress Disorder Related to the COVID-19 Questionnaire (COVID-19-PTSD)

The COVID-19-PTSD [21] is a self-report measure specifically designed to assess post-traumatic stress symptoms (PTSSs) in relation to the COVID-19 pandemic. This questionnaire comprises 19 items referring to experiences within the previous seven days, with responses recorded on a 5-point Likert scale. The internal consistency analysis demonstrated excellent reliability for the selected items (Cronbach’s α = 0.94). In order to achieve the objective of this study, the COVID-19-PTSD was administered in two different versions: (i) a first version that requested a retrospective self-reported evaluation, referring to the first six months of the pandemic; (ii) a second version that requested the current self-reported state according to the questionnaire.

#### 2.2.2. State-Trait Anxiety Inventory (STAI-Y)

The trait scale of the State-Trait Anxiety Inventory (STAI; [32,33]) is a questionnaire designed to evaluate self-reported anxiety on a 4-point Likert scale (1 = not at all; 4 = very much). It assesses both state (current condition) and trait (anxiety as a stable trait) anxiety. Higher scores on the STAI indicate greater anxiety levels. Considering the aim of this study, only trait anxiety (20 items) was assessed. The STAI-Y demonstrated excellent internal consistency of the items (Cronbach’s α values ranging between 0.90 and 0.93).

#### 2.2.3. Emotional Stroop Task

A computerized version of the emotional Stroop task was employed to evaluate executive attention. The target stimuli consisted of colored words (font: Arial; font size: 20; colors: yellow, red, blue, green) that were semantically related to two different categories (type of stimuli: COVID-19; non-COVID-19). Each of them had two different emotional valences (negative, neutral). The stimuli were selected according to an independent validation process. An independent sample of 43 respondents (43% males; mean age = 39.5, SD = 13.5) evaluated 88 words according to emotional valence (from neutral = 0 to negative = 10) and to the association with the COVID-19 pandemic (from not linked to the pandemic = 0 to linked to the pandemic = 10). Table 1 shows the evaluation of each condition of the stimuli included in the emotional Stroop task: (i) COVID-19-related with negative valence; (ii) COVID-19-related with neutral valence; (iii) non-COVID-19 with negative valence; and (iv) non-COVID-19 with neutral valence. Participants were recruited via the dissemination of banners advertising this research through the main social media of the “Sapienza” University of Rome. Of the recruited volunteers, 96 respondents (women: 58.3% of the sample; age range: 19–35; mean age: 24.1; SD = 3.02) met the inclusion criteria of this study (i.e., no diagnosis of COVID-19, no psychopathological or medical conditions, no current medications) and participated in this study. The main characteristics of the sample are shown in Table 2.

The task required participants to respond as quickly and accurately as possible to the color of the ink in which the word on the screen appeared. Participants were instructed to press the key corresponding to the initial letter of the ink color in Italian (key “R” = red; key “V” = green; key “B” = blue; key “G” = yellow). After a brief explanation of the task and a short trial block with stimuli not involved in the experimental procedure, participants completed a block of 160 randomly presented trials (40 for each condition; each word occurs the same number of times for each color). Each trial began with a fixation cross (duration: 500 ms), followed by the presentation of the target stimulus for 2000 ms or until the participant responded. Reaction times (RTs) and proportion of accuracy (number of correct responses/total number of trials) were recorded for the four conditions. The emotional Stroop effect for each type of stimulus was obtained by subtracting the RTs of the neutral trials from the RTs of the negative trials. Two emotional indices (emotional COVID-19, emotional non-COVID-19) were then obtained.

### 2.3. Procedure

The experiment took place in the Laboratory of Health Psychology at the Sapienza University of Rome. Following the signing of the informed consent form and the presentation of this study, each participant completed the survey for the collection of general information, one version of the COVID-19-PTSD (the retrospective or actual version), and the STAI. Then, the emotional Stroop task was administered in a controlled and quiet setting. Finally, participants completed the other version of the COVID-19-PTSD. This study adhered to the principles outlined in the Declaration of Helsinki, and the entire study protocol was approved by the Ethics Committee for Transdisciplinary Research of the Sapienza University of Rome (Resolution No. 87/2023). Participants were permitted to withdraw from this study at any time without providing justification, and no data were retained.

### 2.4. Statistical Analysis

The primary research question of interest was determining the role of COVID-19-related stimuli on attentional bias. To address this question, a mixed 2 × 2 ANOVA was conducted, considering the type of stimuli (COVID-19/non-COVID-19) and the valence of the stimuli (negative/neutral) on both reaction times and proportion of accuracy. Moreover, an ANOVA considering the emotional Stroop effect in COVID-19 and non-COVID-19 trials was conducted. To assess whether psychological dimensions may affect emotional attentional processing, regression models were fitted for each condition of the emotional Stroop task (negative COVID-19, neutral COVID-19, negative non-COVID-19, neutral non-COVID-19). The model included as predictors the retrospective self-reported COVID-19-PTSD score, the current levels of self-reported COVID-19-PTSD, the STAI score, and sex. For all statistical analyses, significance was set at *p* < 0.05. The analyses were conducted using Statistica software (version 10.0, Dell, Round Rock, TX, USA).

## 3. Results

The ANOVA on the reaction times of the emotional Stroop task did not reveal a significant effect for the type of stimuli (COVID-19; non-COVID-19; F1,4 = 2.01; *p* = 0.16), for the valence (negative; neutral; F1,94 = 1.38; *p* = 0.25), nor the type of stimuli x valence interaction (F2,94 < 1; *p* = 0.37).

ANOVA on the proportion of accuracy showed higher accuracy in identifying COVID-19-related stimuli than non-COVID-19 stimuli (F1,94 = 4.78; *p* = 0.03). However, no other significant effects were highlighted (all F < 1). For further details, see Table 3.

### Regression Models

The single predictive models for each condition of the emotional Stroop task (see Table 3) showed that the models were significant for COVID-19-related stimuli in both negative and neutral valence. Specifically, for negative valence, the four predictors accounted for 14% of the total variance in the outcome (F 4, 94 = 3.14, *p* = 0.02). Faster reaction times for COVID-19-related negative stimuli were significantly associated with higher retrospective COVID-19-PTSD (b = −1.51, SE = 0.57, t = −2.62, *p* = 0.01) and in females (b = 34.83, SE = 13.91, t = 2.50, *p* = 0.01). (see Table 3). Also, for COVID-19-related neutral stimuli, the four predictors accounted for 13% of the total variance in the outcome (F 4, 94 = 2.79, *p* = 0.02). Faster reaction times for COVID-19-related neutral stimuli were significantly associated with higher retrospective COVID-19-PTSD (b = −1.25, SE = 0.61, t = −2.05, *p* = 0.04) and in females (b = 40.65, SE = 14.82, t = 2.74, *p* = 0.01). (see Table 4).

## 4. Discussion

The global impact of the COVID-19 pandemic was undoubtedly profound and multifaceted, particularly in terms of mental health, behavioral patterns (i.e., sleep [34,35]), and social interactions (e.g., [17,36,37]). Individuals exhibited heightened sensitivity to any event that could be linked back to the pandemic, although this sensitivity manifested in varying degrees of severity across the globe. From this premise, our study investigated cognitive responses to threatened or possibly threatening stimuli in the post-COVID-19 landscape, comparing old and new concerns. The importance of delineating the manner in which individuals respond to negative stimuli, particularly those associated with stressful situations, has been extensively reported in the literature. This response is thought to be associated with the role of attentional bias in the development of maladaptive coping mechanisms in response to negative life experiences [38,39].

Consequently, it is important to verify whether and to what extent cognitive responses are still affected by the traumatic resonance of the pandemic. This is relevant for understanding how significantly an unexpected and sudden event can influence long-term perspectives. Conversely, this study may provide direct evidence regarding the similarities and differences in stimuli, the elaboration of negative emotional stimuli, and the specific and direct experiences of events that have been overexposed in the media.

The results indicate that high selective attention to COVID-19-related stimuli, as evidenced by faster reaction times at the emotional Stroop task for both neutral and negative valence, was consistently correlated with higher retrospective COVID-19-PTSS but not with current concerns. To elucidate these findings, it is crucial to revisit the events during the pandemic’s onset. The unprecedented changes, such as lockdowns, social distancing measures, and economic disruptions, have led to elevated stress, anxiety, and subsequent PTSD worldwide [21,40,41]. Our previous study reported a significant increase in mental distress and the emergence of COVID-19-PTSS, estimating an increase during the first months of the pandemic [17,20,22,34,35], as similarly reported by our retrospective data in this study. This surge can be attributed to a number of factors, including fear of infection, grief from the loss of loved ones, and uncertainty about the future [42,43,44]. However, as many authors have suggested, the extent to which this is the case may depend on the high dissemination of news and the worrying tone adopted by the media, which have caused an increase in concern among the population. The present study also suggests a moderate attentional bias toward COVID-19-related stimuli. Specifically, although no differences in reaction times were observed in the performance of the task, individuals were more accurate in responding to COVID-19-related stimuli. This indicates a tendency for attention to be drawn towards these stimuli when presented alongside non-COVID-19-related stimuli. This tendency is consistent with the fact that selective attention is powerfully biased toward threat-related information as an evolutionarily adaptive response in environments where dangers constantly threaten survival and reproductive advantage [45,46]. However, it is notable that the valence of the stimuli did not influence the performance, suggesting that we should reflect on the role of threat in attentional processing. This implies that all COVID-19-related stimuli have assumed greater salience, regardless of their valence. This also provides an interesting hypothesis regarding the potential influence of overstimulation with COVID-19-related stimuli rather than the threat component itself. Given the timing of this study, it is surprising that this effect was observed across the sample. An intriguing finding is that experiential and demographic factors influenced the salience of COVID-19-related stimuli, resulting in a varied pattern of attentional bias. Specifically, we found that variables such as sex and experience of PTSS related to COVID-19 were significant predictors of attentional bias.

These variables would be the causes of the increased sensitivity to negative stimuli associated with COVID-19 [29]. Research indicates that continuous exposure to distressing aspects of the pandemic exacerbated anxiety and depression [41,47]. Also, Holmes et al. [48] highlighted that the pervasive sense of threat and uncertainty during the pandemic heightened alertness and fear [49,50,51]. In this context, as previously suggested by our studies, the pandemic-related symptomatology associated with COVID-19-related post-traumatic stress disorder was associated with deficits in inhibiting preponderant responses, indicating an executive function deficit affecting goal-directed actions in general, particularly in at-risk populations [9]. Moreover, as reported by Cannito’s study [52], an increase in anxiety levels would result in an attentional bias toward virus-related stimuli, influencing perceptions of future consequences [52]. Additionally, individuals experiencing higher pandemic-related stress showed lower attentional bias towards COVID-19-related information in the presence of higher levels of alexithymia [53]. Our findings support and reinforce these observations, highlighting that responses to COVID-19 stimuli, irrespective of valence and whether or not they are activating, reflect genuine reactions to the stimulus itself, shaped more by concerns during the pandemic period than current worries about the virus, as well as a general personological trait of anxiety. This suggests that COVID-19-related distress has been reduced, as evidenced by the reduction in PTSS scores reported by participants (differences in the scores between retrospective and current PTSS). It also indicates that past experiences related to the pandemic have had a lasting impact on perceptions and reactions. Finally, it highlights the long-term psychological and behavioral effects of past emergencies.

In general, the results of this study should be interpreted within the context of a comprehensive theoretical and empirical framework. While there is a lack of specific models that elucidate how attentional processes interact with COVID-19 stimuli to influence future mental health concerns, insights from the related literature are valuable. For example, research on anxiety disorders suggests that attentional capture by threatening stimuli facilitates extensive information processing [54,55]. Similarly, studies on depression suggest that heightened attention to sad stimuli may sustain chronic depressive symptoms through rumination [56]. Building on these frameworks, we propose that increased attention to COVID-19-specific terms may activate negative emotions and thoughts associated with pandemic-related losses, potentially impairing conflict resolution abilities by taxing executive functions [57]. Given the extensive media coverage of the COVID-19 pandemic, biased attention towards pandemic-related content could reinforce negative associations with loss, potentially disrupting the grieving process. It is possible that repeated exposure to COVID-19 stimuli may cumulatively sustain elevated distress levels, increasing the risk of subsequent mental health issues. This cognitive response may be associated with continual exposure to distressing information, which may lead to cognitive overload, impaired decision-making, reduced flexibility, and increased susceptibility to misinformation [58,59,60,61,62,63]. The phenomenon of “Doomscrolling”, i.e., the compulsive consumption of negative news, has become prevalent during the pandemic. Garfin, Silver, and Holman [64] discussed how constant exposure to alarming news creates a feedback loop of negative emotions, reinforcing feelings of dread and helplessness. This behavior exacerbates existing mental health issues and contributes to the development of new psychological problems. Liu [65] found a direct correlation between the frequency of COVID-19 news consumption and increased psychological distress among young adults, indicating that higher engagement with pandemic-related news predicts symptoms of anxiety, depression, and distress related to COVID-19 (e.g., PTSS, PTSD).

Although these new insights into the role of the pandemic in individual functioning are important, it is important to acknowledge some limitations of this research. Firstly, the cross-sectional nature of this study precludes the possibility of drawing definitive conclusions regarding the direct influence of the COVID-19 pandemic on attentional processes, despite the use of retrospective inquiries. Additionally, the use of the emotional Stroop task in this study may influence the interpretations of attentional patterns due to the large debate regarding the optimal interpretation of its outcomes. Specifically, different perspectives have suggested different interpretations that consider the outcome of the Stroop task as indices of attentional control, cognitive inhibition, or other attentive processes (e.g., [66,67]).

Another limitation of this study is the administration of two different versions of the COVID-19-PTSD: (i) retrospective self-reported traumatic experience with the COVID-19 pandemic and (ii) current distress associated with COVID-19. The retrospective version may be subjected to memory biases and should be carefully considered as an index of the real PTSS perceived during the emergency phase. However, considering previous studies the indication emerging from our study may provide reliable insight about the role of past self-reported distress. Finally, it is important to extend these studies to multiple populations and contexts (what about healthcare professionals who were the most burdened by the pandemic experience?).

## 5. Conclusions

In conclusion, despite these limitations, our findings indicate that attentional biases may be a significant consequence of the COVID-19 pandemic, warranting further investigation in the literature. These findings provide a rationale for future research into attentional bias modification strategies that may alleviate mental health concerns and eventually influence the behaviors of those who experienced severe psychological consequences after the emergency period. Together, these findings suggest that the association between COVID-19 stimuli and attentional biases extends beyond emotional valence, being retrospectively influenced by mental health during the pandemic, pointing towards pathways to future mental health challenges. It may be beneficial to assess additional aspects of attentional control and their interactions with other attentional networks (e.g., [68]).

## Figures and Tables

**Table 1:** Main characteristics of the sample.

	Mean (SD)	CI 95%
Age	24.1 (3.02)	23.4–24.7
Years of education	17.6 (2.10)	17.1–18.0
Trait anxiety (STAI)	47.5 (11.00)	45.1–49.9
Retrospective COVID-19-PTSS *	22.3 (13.60)	19.3–25.3
Current COVID-19-PTSD	6.4 (10.00)	4.19–8.61

* % Percentage of critical score at the COVID-19-PTSD: 28 (34.1%). CI: confidence interval; PTSD: post-traumatic stress disorder; SD: standard deviation.

**Table 2:** Mean and standard deviation (SD) of the stimuli adopted in the emotional Stroop task.

	COVID-19-Related Stimuli		Non-COVID-19 Stimuli	
	Negative	Neutral	Negative	Neutral
COVID-19 Association	8.60 (1.34)	9.40 (0.90)	1.0 (0.71)	0.60 (0.55)
Valence	8.60 (0.55)	2.80 (0.84)	9.80 (0.45)	1.40 (0.55)
Words	Virus, Pandemia, Morti, Reclusione, Polmonite,	Disinfettante, Mascherina, Decreto, Tampone, Vaccino,	Guerra, Bombardamento, Cancro, Terremoto, Omicidio,	Raccoglitore, Termosifone, Triangolo, Etichetta, Muro,
English translation	Virus, Pandemic, Deaths, Reclusion, Pneumonia	Disinfectant, Mask, Decree, Swab, Vaccine	War, Bombing, Cancer, Earthquake, Murder	Binder, Radiator, Triangle, Label, Wall

**Table 3:** Descriptive statistics of COVID-19 and non-COVID-19-related stimuli.

	COVID-19-Related Stimuli				Non-COVID-19 Stimuli			
	Negative		Neutral		Negative		Neutral	
	M (SD)	CI 95%	M (SD)	CI 95%	M (SD)	CI 95%	M (SD)	CI 95%
Reaction Time	626.0 (68.4)	612–640	625 (68.9)	611–639	632 (65.6)	618–645	626 (63.4)	613–619
Proportion of accuracy	0.97 (0.03)	0.96–0.97	0.97 (0.03)	0.96–0.98	0.96 (0.04)	0.95–0.97	0.96 (0.04)	0.95–0.97
Emotional effects	0.78 (34.4) [−6.31–7.11]				5.40 (37.2) [−2.19–3.13]			

CI: confidence interval; SD: standard deviation.

**Table 4:** Summary of regression analyses.

	COVID-19-Related Stimuli		Non-COVID-19 Stimuli	
	Negative	Neutral	Negative	Neutral
R	0.37	0.35	0.29	0.30
R^2^	0.14	0.13	0.09	0.10
F	3.14	2.79	1.85	2.39
*p*	0.02	0.03	0.13	0.06

## Data Availability

The data presented in this study are available on request from the corresponding author due to privacy restrictions.
